# Effect of Five-Consecutive-Day Exposure to an Anxiogenic Stressor on Sleep-Wake Activity in Rats

**DOI:** 10.3389/fneur.2013.00015

**Published:** 2013-02-26

**Authors:** Matthew W. O’Malley, Rachel Lea Fishman, Domenic A. Ciraulo, Subimal Datta

**Affiliations:** ^1^Laboratory of Sleep and Cognitive Neuroscience, Department of Psychiatry, Boston University School of MedicineBoston, MA, USA; ^2^Department of Neurology, Boston University School of MedicineBoston, MA, USA; ^3^Program in Behavioral Neuroscience, Boston University School of MedicineBoston, MA, USA

**Keywords:** anxiety, depression, sleep-wake, rat model, foot-shock

## Abstract

Repeated exposure to an anxiogenic stressor (AS) is a known environmental factor for the development of depression, yet the progression of sleep-wake (S-W) changes associated with the onset of AS-induced depression (ASID) is not completely understood. Thus, the aim of this study was to identify these progressive S-W changes by developing ASID in rats, via repeated exposure to an AS, and compare this ASID-associated sleep phenotype with the sleep phenotype of human depression. To achieve this aim, rats were first recorded for a 6 h period of baseline S-W activity without AS. Then, rats were subjected to 5 days of AS [Day 1: inescapable foot-shock; 5 trials of 3 s foot-shocks (1.0 mA) at 3 min intervals; Days 3–5: 15 trials of 5 s foot-shocks at 45 s intervals]. S-W activity was recorded for 6 h immediately after each AS treatment session. Two days later rats were again recorded for 6 h of S-W activity, but with no exposure to the AS (NASD). Compared to the baseline day: Day 1 of AS (ASD-1) increased wakefulness, slow-wave sleep (SWS) latency, and rapid-eye movement (REM) sleep latency, but decreased the total amount of REM sleep; ASD-2 animals remained awake throughout the 6 h S-W recording period; ASD-3, ASD-4, and ASD-5 (ASDs-3–5) decreased wakefulness, SWS latency, and REM sleep latency, but increased the total amount of REM sleep. Interestingly, these results reveal that initial exposure to the AS versus later, repeated exposure to the AS produced opposing S-W changes. On NASD, animals exhibited baseline-like S-W activity, except slightly less REM sleep. These results suggest that repeated AS produces a sleep phenotype that resembles the sleep phenotype of depression in humans, but consistent re-exposure to the AS is required. These results are promising because the methodological simplicity and reversibility of the ASID-associated S-W phenotype could be more advantageous than other animal models for studying the pathophysiological mechanisms that underlie the expression of ASID.

## Introduction

Endogenous depression is a debilitating mental health disorder that currently affects about 121 million people worldwide, contributing to the loss of approximately 850,000 lives each year (http://www.who.int/mental_health/management/depression/definition/en/). It is characterized by a multitude of symptoms that have emotional, behavioral, and physical aspects (Fawcett and Kravitz, 1983). The most common emotional symptoms include depressed mood, which persists for at least 2 weeks; loss of interest in pleasurable activities; feelings of worthlessness or guilt; and recurrent thoughts of death or suicide (American Psychiatric Association, 2000). The most common cognitive symptoms in depressed patients are difficulty thinking, concentrating, and decision-making (Ciraulo and Shader, 2011). The most common physical symptoms are altered sleep, disruption of circadian rhythms, fatigue, pain and discomfort, psychomotor agitation or retardation, and appetite changes (Gerber et al., 1992). Since depression impacts all body systems, it is not surprising that investigators have attempted to determine the effects of depression on many biological markers, including sleep architecture, hormones, neurotransmission, brain imaging, and immune function in order to identify the underlying neuropathology of depression (Coble et al., 1976; Kupfer, 1976, 1978; Gillin et al., 1979; Reynolds and Kupfer, 1987; Kroenke and Price, 1993; Sharpley and Cowen, 1995; Posse and Hällström, 1998; Ciraulo and Shader, 2011; Goldstein et al., 2012).

There are many therapeutic options for treating depression such as psychotherapy (i.e., interpersonal, psychodynamic, and cognitive behavioral therapy; Buchheim et al., 2012), drug treatment (i.e., selective serotonin reuptake inhibitors, serotonin norepinephrine reuptake inhibitors, norepinephrine-dopamine reuptake inhibitors, monoamine oxidase inhibitors, and tricyclics; Guelfi et al., 1992; Frazer, 1997; Pinder, 1997; Matreja et al., 2012), physical procedures (i.e., electroconvulsive therapy, Vagus nerve stimulation, acupuncture, and repetitive transcranial magnetic stimulation; Rush et al., 2005; Allen et al., 2006; Rachid and Bertschy, 2006; Gaynes et al., 2011; Martinez-Amoros et al., 2012), and general life changes (i.e., improvements in diet, exercise, and sleep; Bountziouka et al., 2009; Vallance et al., 2011). However, the efficacy of these treatment options has been shown to vary greatly, which warrants a better understanding of the etiology and pathophysiological mechanisms of depression and its associated symptoms.

To better understand the pathophysiological mechanisms that underlie these sleep-wake (S-W) disturbances, it is important to identify an animal model of depression that emulates the sleep phenotype commonly observed in humans with depression. In order to achieve this understanding, a number of rat models have been developed that simulate both endogenous (genetic and brain chemistry alterations; e.g., neonatal drug administration, genetic alteration) and exogenous [environmental factors; e.g., parental separation, learned helplessness (LH)] depression (Jesberger and Richardson, 1985; Willner, 1990; Minor et al., 1994a,b; Dugovic et al., 2000; Mavanji et al., 2002; Kinn et al., 2008). However, none of these models have been used previously to study the progression of S-W disturbances in depressed animals. Also, these animal models have inherent constraints such as an elongated experimental timetable, borderline ethical justification (excessive number, duration, and intensity of foot-shock), and an irreversibility (depression cannot be eradicated once depression is acquired). Therefore, the goal of the present study was to develop an animal model of depression that circumvents these constraints.

Recurring exposure to an anxiogenic stressor (AS) is a major causal factor of depression (anxiogenic stress-induced depression; ASID). Numerous studies have utilized repetitive inescapable foot-shock as an AS to induce depression using a range of number, duration, and intensity of foot-shocks. Thus, in the present study, the chosen foot-shock parameters were strong enough to induce depression and a reversible depression-associated S-W phenotype, but not so strong that they bordered on any ethical boundaries, which have become increasingly stringent in recent years, as occurs in LH studies (Adrien et al., 1991; Fogel et al., 2011). Additionally, experimental testing was conducted in adult rats, which drastically reduced total experimental duration, compared to some of the aforementioned animal models of depression. Also, unlike LH which calls for a learning component to be used for assessment of acquired depression, the ASID model used in the present study incorporates no such component, thereby negating any possible effect of learning (beyond the inherent foot-shock-associated learning) on the desired ASID-associated S-W changes. The S-W findings of this study suggest that the method we used to induce ASID in experimental animals indeed produced a sleep phenotype resembling that of human depression. A future study utilizing this model and incorporating an avoidance learning component could then be conducted to confirm the ASID model used in this study does indeed produce LH, and if confirmed, this model could then also be used to study the pathophysiological mechanisms underlying sleep disturbances in depression.

## Materials and Methods

### Subjects and housing

Experiments were performed on 12 adult male Wistar rats (Charles River Laboratories, Wilmington, MA, USA) weighing between 325 and 400 g. These rats were housed individually at 22°C, with *ad libitum* access to food and water, under 12 h light cycle (7:00 a.m. to 7:00 p.m.)/12 h dark cycle (7:00 p.m. to 7:00 a.m.) conditions. The principles for the care and use of laboratory animals in research as outlined by the National Institutes of Health *Guide for the Care and Use of Laboratory Animals* (1996) were strictly followed. Additional care was taken to ensure that any potential discomfort, as well as the number of animals used, was minimal. Also, in order to reduce potential increases in stress imposed by experimental handling, animals were handled daily for 15–20 min between 9:00 a.m. and 10:00 a.m. These habituation handling sessions began 1 week prior to surgery and continued up until the day of surgery.

### Surgical procedure for electrode implantation

All surgical procedures were performed stereotaxically under aseptic conditions in accordance with the guidelines approved by the Institutional Animal Care and Use Committee. Animals were first anesthetized with sodium pentobarbital (40 mg/kg, i.p.; Ovation Pharmaceuticals, Deerfield, IL, USA), then placed in the stereotaxic apparatus using blunt rodent ear bars and secured in the flat-skull position, as described previously (Paxinos and Watson, 1998). The appropriate depth of anesthesia was judged by the absence of palpebral reflexes and a response to tail pinch. Core body temperature was maintained at 37 ± 1°C with a thermostatic heating pad and a rectal thermistor probe. A scalp incision was made, and the skin was retracted. The skull surface was then cleaned in preparation for electrode implantation. Upon completion of the surgical procedure, ampicillin (50 mg/rat, s.c. Bristol-Meyers Squibb, Princeton, NJ, USA) was administered to control potential post-surgical infection and buprenorphine (0.03 mg/kg s.c.; Ben Venue Laboratories, Bedford, OH, USA) was administered to control potential post-operative pain.

To record vigilance states, cortical electroencephalogram (EEG; to record cortical activity), dorsal neck muscle electromyogram (EMG; to record neck muscle activity), and hippocampal EEG (to record theta wave activity) recording electrodes were implanted, as described in our earlier publications (Datta, 2000; Datta et al., 2008). Immediately after surgery, animals were placed in recovery cages and monitored for successful recovery from anesthesia and surgery. Upon successful recovery, gaged by the return of normal postures, voluntary movement, and grooming, animals were returned to their normal housing.

### Habituation to sleep-wake recording conditions

After a post-surgical recovery period of 7–10 days, rats underwent a 7 day habituation period to a sound-attenuating polygraphic S-W recording cage (electrically shielded: 76.2 × 45.7 × 45.7 cm) under free-moving conditions. The habituation period featured daily 6 h sessions (10:00 a.m. to 4:00 p.m.) in the recording cage, which also served as baseline recording sessions for electrode testing and monitoring of daily variations in the percentages of time spent in different S-W states. Additionally, during the recovery and habituation periods, rats continued to experience a 12 h light/12 h dark cycle with free access to food and water.

### Anxiogenic stressor procedure

A freezing chamber was used as the experimental apparatus. This chamber was constructed of acrylic side walls and a Plexiglas front wall, and included a floor consisting of stainless steel bars, which were wired to a shock source and solid-state grid scrambler for the delivery of foot-shocks. In the present study, the day before ASID treatment began was also the final day of habituation, and the S-W recording from this day was used as the baseline S-W recording (BLD). Starting the following day, inescapable foot-shock was used for the induction of anxiety over a span of 5 days. At the beginning of each day, naïve rats were transported from their housing room in a clear plastic cage to the experimental testing room. On ASD-1, within a minute upon arrival, rats were placed inside the testing chamber (at approximately 9:40 a.m.) and were allotted 2 min of adaptation time. This was followed by five trials of 3 s scrambled foot-shocks (1.0 mA), which occurred in fixed intervals of 3 min, for a total of 14 min (between 9:40 a.m. and 9:55 a.m.). On ASDs-3–5, rats were again placed in the freezing chamber and allotted a 2 min adaptation period. Rats then received 15 trials of 5 s foot-shocks (1.0 mA once again) at 45 s intervals, for a total of approximately 14 min (between 9:40 a.m. and 9:55 a.m.). On each day, at the conclusion of each foot-shock session, rats were removed from the testing chamber and connected to a polygraphic recording system, where S-W activities were recorded for 6 h (10:00 a.m. to 4:00 p.m.). Two days after the final foot-shock session, rats were recorded for 6 h of undisturbed S-W activity (10:00 a.m. to 4:00 p.m.), but with no exposure to the anxiogenic stressor (NASD). Additionally, the testing chamber was cleaned with chlorine dioxide disinfectant (MB-10, Quip Laboratories, Wilmington, DE, USA) before and after each use.

### Analysis of behavioral states and cortical EEG power spectra

For the purpose of gaging potential changes in S-W activity, polygraphic data were captured on-line with a computer using Gamma software (Grass product group; Astro-Med, West Warwick, RI, USA). From this captured data, four behavioral states were distinguished and scored visually using SleepSign for Animal software (Kissei Comtec Co., Ltd, Tokyo, Japan): W, slow-wave sleep (SWS), transitional sleep between SWS and REM sleep (tS-R), and rapid-eye movement (REM) sleep. The physiological criteria for the identification of these S-W states have been described in detail previously (Datta and Siwek, 2002; Datta et al., 2004; Thakkar et al., 2008), and the scoring of these states was conducted in successive 5 s epochs. The polygraphic measures provided the following dependent variables that were quantified for each animal: (1) percentage of recording time spent in W, SWS, tS-R, and REM sleep; (2) latencies to the onset of SWS and REM sleep; and (3) total number of REM sleep episodes. The effect of shock treatment on the percentages of time spent in W, SWS, tS-R, and REM sleep were statistically analyzed using a two-way ANOVA followed by Bonferroni post tests, with time as a repeated-measure variable (six levels corresponding to the six 1 h epochs of the 6 h S-W recording) and treatment as a between-subjects variable (six levels corresponding to the six different test days, BLD, ASD-1, ASDs-3–5, and NASD). On ASD-2, animals did not sleep at all during the 6 h S-W recording period, therefore, S-W data was not used for statistical analyses (thus, ASD-2 S-W data is not included in any figures in this manuscript). One-way ANOVA and appropriate *post hoc* *t*-tests were then performed to determine the individual levels of significant difference in rats for each treatment day. Similar analyses were used to analyze the latencies for SWS and REM sleep as well as the total number of REM sleep episodes. These collective statistical tests were performed using Prism software (GraphPad Software, Inc., La Jolla, CA, USA).

For power spectral analysis, cortical EEG signals were amplified and bandpass filtered (0.2–100 Hz) with a polygraph and Gamma software, as described in our earlier publications (Maclean and Datta, 2007; Huang et al., 2010). The amplified and filtered data was digitized at a sampling frequency of 200 Hz and subjected to a Fast Fourier transformation (SleepSign software) to calculate the cortical EEG power during the total 6 h recording. Analysis focused on two frequency ranges: delta (0.2–4.0 Hz) and theta (5–10 Hz). The power of each frequency band was averaged and expressed as the percentage of total power within the frequency range of 0.2–40 Hz. These two variables were then analyzed using two-way ANOVA followed by Bonferroni post tests (Prism software).

## Results

### Effects on sleep-wake architecture

**Figure 1:**
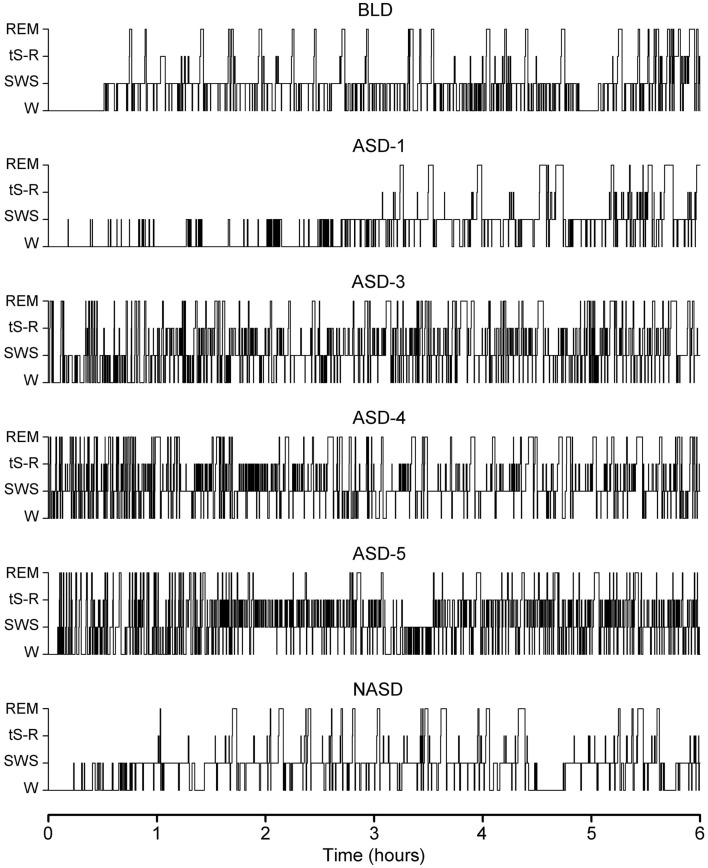
**Progression of sleep-wake architecture that occurred over the course of the six experimental test days, listed chronologically: no exposure to anxiogenic stressor (AS) baseline day (BLD), day 1 of AS exposure (ASD-1), day 3 of AS exposure (ASD-3), day 4 of AS exposure (ASD-4), day 5 of AS exposure (ASD-5), and no exposure to AS day (NASD)**. Each of the six hypnograms is an example of the sleep-wake architecture expressed by rats during the 6 h sleep-wake recording period that immediately followed that experimental treatment. These continuous step hypnograms plot occurrence and duration of polygraphically and behaviorally defined wakefulness (W), slow-wave sleep (SWS), transitional sleep between SWS and REM sleep (tS-R), and REM sleep.

Figure 1 shows the representative S-W architectures of a single rat for the 6 h recordings (10:00 a.m. to 4:00 p.m.) that immediately followed baseline, shock, and no shock session. These S-W architectures show that ASD-1 produced a massive increase in REM sleep latency and fewer REM sleep episodes compared to the baseline. Also, SWS episodes in the first 3 h were short and sporadic, and entry into a relatively continuous level of sleep similar to that seen on the BLD did not occur until much later in the recording. Interestingly, later in the recording, especially during the 4 and 5 h periods, the duration of SWS episodes was notably greater in ASD-1 than in the BLD.

ASDs-3–5 of shock treatment were fairly similar and each had the inverse effect on REM sleep from ASD-1. There was a drastic reduction in REM sleep latency and massive increase in the number of both tS-R and REM sleep episodes, particularly in the first 2 h of recording, compared to the BLD. Also, the duration of REM sleep episodes was very short during the first 3 h or so of the recording session but grew consistently longer in the final 3 h of recording, which more closely resembled the REM sleep durations of the BLD and ASD-1.

No exposure to the anxiogenic stressor produced little-to-no observable effect on the overall S-W architecture compared to the BLD. Specifically, SWS latencies, REM sleep latencies, and the number of REM sleep episodes were all comparable. The collective S-W architectures strongly indicate that the exposure to inescapable shock drastically impacted the normal S-W activity in rats, and that this impact was two-fold – ASD-1 had one effect, and ASDs-3–5 produced a much different effect. Also, the lack of stressor exposure seemed to strongly benefit the rats, as their S-W activity returned relatively close to BLD levels.

### Effects on wakefulness

**Figure 2:**
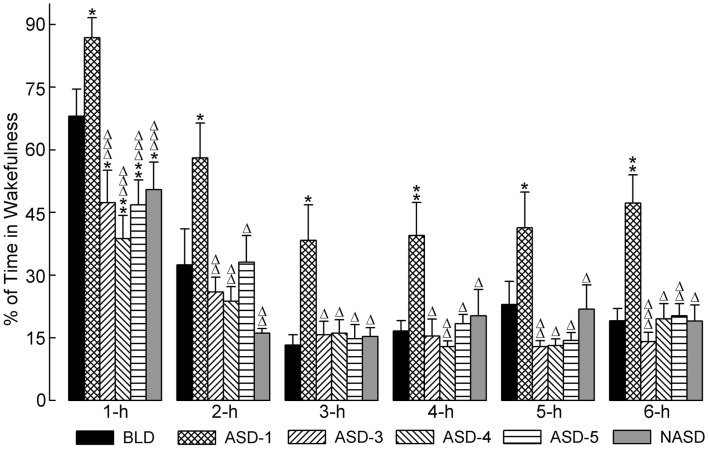
**Progressive effects of repetitive anxiogenic stressor (AS) exposure on the percentages (mean ± SEM) of time spent in wakefulness for each hour of the 6 h sleep-wake recording period immediately following experimental treatment**. The treatments, listed chronologically: no exposure to AS baseline day (BLD), day 1 of AS exposure (ASD-1), day 3 of AS exposure (ASD-3), day 4 of AS exposure (ASD-4), day 5 of AS exposure (ASD-5), and no exposure to AS day (NASD). *Represents a significant difference from BLD: *p < 0.05, **p < 0.01, ***p < 0.001. Δ represents a significant difference from ASD-1: Δ*p* < 0.05, ΔΔ*p* < 0.01, ΔΔΔ*p* < 0.001.

Figure 2 shows the effects of shock treatment on the percentages of time spent in W. A two-way ANOVA indicated a significant main effect of treatment [*F*(4, 225) = 11.30, *p* < 0.001], time [*F*(5, 225) = 76.79, *p* < 0.001], and treatment × time interaction [*F*(20, 225) = 2.812, *p* < 0.05]. Also presented in Figure 2 are the results of *post hoc* analyses (*t*-tests) on W%. These analyses revealed that, compared to the BLD, the amount of time spent in W was significantly greater on ASD-1, but significantly less on ASDs-3–5, which were comparable with each other. Additionally, the significant difference in W% between ASD-1 and the BLD was evident throughout the entire 6 h of recording, while the differences between ASDs-3–5 with the BLD were only present in the 1 h period. Also, W% on ASDs-3–5 was significantly reduced from that of ASD-1, and this deviation was exhibited throughout the entire 6 h recording. Collectively, these results indicate that stressor exposure imparted a significant effect on the amount of time spent in W, but that this effect triggered two distinct changes in the amount of W.

*Post hoc* comparisons involving the NASD yielded results similar to those of ASDs-3–5. Specifically, there was a significant reduction in W% compared to the BLD, which was present only in the 1 h period, and there was also a significant reduction in W% compared to ASD-1, which was evident throughout the entire 6 h recording. The close similarity between the NASD with ASDs-3–5 indicates that the final three consecutive days of shock treatment imparted a lasting effect on the amount of time spent in W, and that 2 days of relief from stressor exposure is not enough to undo this effect.

### Effects on slow-wave sleep

**Figure 3:**
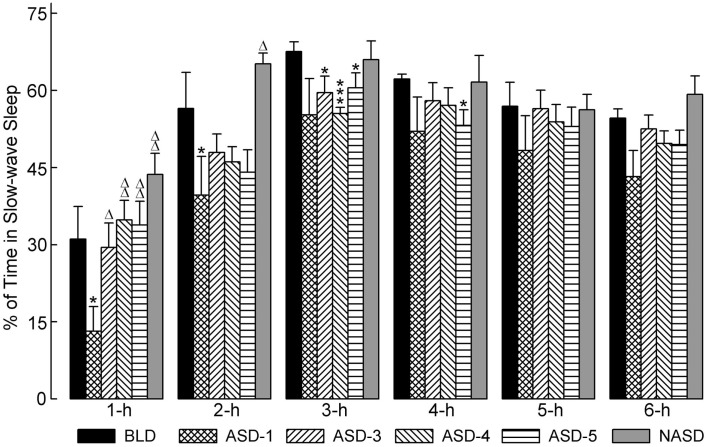
**Progressive effects of repetitive anxiogenic stressor (AS) exposure on the percentages (mean ± SEM) of time spent in slow-wave sleep for each hour of the 6 h sleep-wake recording period immediately following experimental treatment**. The treatments, listed chronologically: no exposure to AS baseline day (BLD), day 1 of AS exposure (ASD-1), day 3 of AS exposure (ASD-3), day 4 of AS exposure (ASD-4), day 5 of AS exposure (ASD-5), and no exposure to AS day (NASD). *Represents a significant difference from BLD: *p < 0.05, **p < 0.01, ***p < 0.001. Δ represents a significant difference from ASD-1: Δ*p* < 0.05, ΔΔ*p* < 0.01, ΔΔΔ*p* < 0.001.

Figure 3 illustrates the effects of shock administration on the percentages of time spent in SWS per hour. A two-way ANOVA indicated a significant main effect of time [*F*(5, 225) = 51.15, *p* < 0.001] only. Also presented in Figure 3 are the results of *post hoc* analyses (*t*-tests) on SWS%. These results show that the percentage of time spent in SWS on ASD-1 was significantly reduced during the 1 and 2 h periods compared to the BLD, and significantly reduced during the 1 h period compared to ASDs-3–5. Also, SWS% was markedly reduced in the 2 h period and significantly reduced in the 3 h period on ASDs-3–4, as well as in the 4 h period on ASD-5, compared to the BLD (ASDs-3–5 were comparable to each other, similar to W%). These collective results suggest that shock treatment produced deficits in the amount of time spent in SWS, but that this deficit was expressed early on in the recording on ASD-1 and during the middle of recording on ASDs-3–5.

*Post hoc* comparisons with the NASD revealed that the amount of time spent in SWS was comparable to that of the BLD throughout the 6 h recording. Also, SWS% was significantly greater in the 1 and 2 h periods on the NASD compared to ASD-1. These results suggest that 2 days of relief from shock exposure is enough to alleviate the ASID-associated disturbances to SWS.

### Effects on transitional sleep

**Figure 4:**
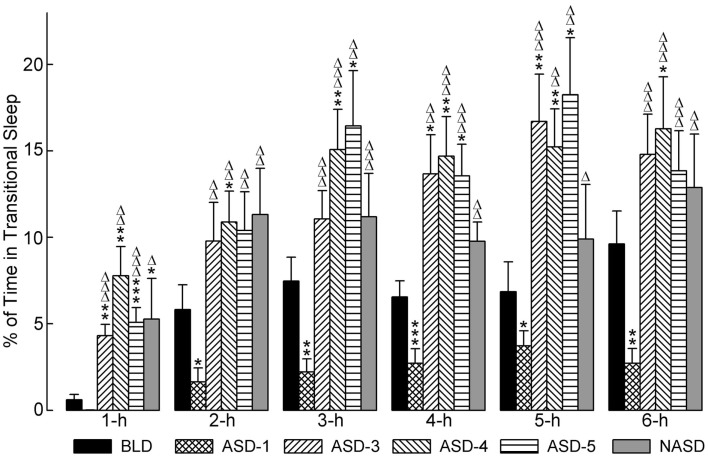
**Progressive effects of repetitive anxiogenic stressor (AS) exposure on the percentages (mean ± SEM) of time spent in transitional sleep between slow-wave sleep and REM sleep for each hour of the 6 h sleep-wake recording period immediately following experimental treatment**. The treatments, listed chronologically: no exposure to AS baseline day (BLD), day 1 of AS exposure (ASD-1), day 3 of AS exposure (ASD-3), day 4 of AS exposure (ASD-4), day 5 of AS exposure (ASD-5), and no exposure to AS day (NASD). *Represents a significant difference from BLD: *p < 0.05, **p < 0.01, ***p < 0.001. Δ represents a significant difference from ASD-1: Δ*p* < 0.05, ΔΔ*p* < 0.01, ΔΔΔ*p* < 0.001.

Figure 4 represents the effects of shock and no shock sessions on the percentages of time spent in tS-R per hour. A two-way ANOVA indicated a significant main effect of treatment [*F*(4, 225) = 10.80, *p* < 0.0001], time [*F*(5, 225) = 31.26, *p* < 0.0001], and treatment × time interaction [*F*(20, 225) = 1.913, *p* = 0.0127]. The detailed results of *post hoc* analyses (*t*-tests) on the total percentages of time spent in tS-R are also presented in Figure 4. These results revealed a marked reduction in the percentage of time spent in tS-R in the 1 h period on ASD-1 compared to the BLD, and significant reductions in the 2, 3, 4, 5, and 6 h periods. Also, tS-R% values on ASDs-3–5 were comparable (similar to both W% and SWS%), and these tS-R% values were significantly increased, compared to the BLD and ASD-1, throughout the 6 h recording. These results denote the large impact of shock treatment on tS-R%, specifically the differential effect between first-time stressor exposure, which caused a sharp reduction in tS-R% (signifying an absence of homeostatic drive for REM sleep), and subsequent repeated exposure, which produced a massive increase in tS-R% (signifying an increased homeostatic drive for REM sleep).

*Post hoc* comparisons involving the NASD revealed that tS-R% was significantly higher on the NASD compared to ASD-1 throughout the entire 6 h recording (similar to ASDs-3–5). However, compared to the BLD, tS-R% was significantly higher during the 1 h period, but then comparable for the remaining 5 h of recording (dissimilar to ASDs-3–5). These collective results suggest that 2 days of relief from shock treatment is enough for tS-R to begin to return to normal baseline levels.

### Effects on REM sleep

**Figure 5:**
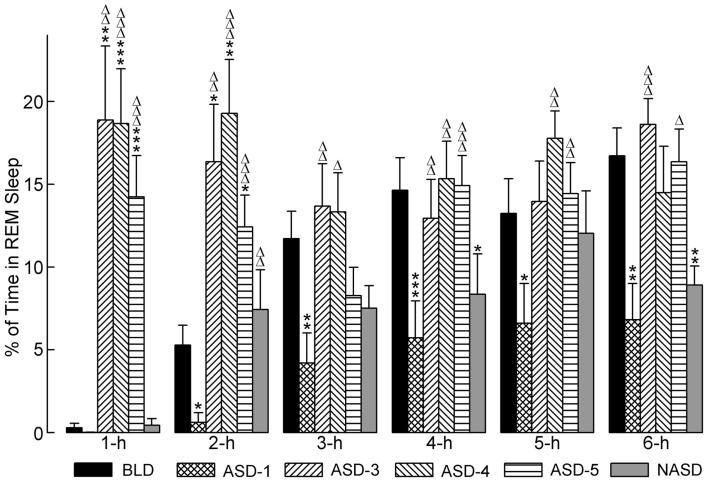
**Progressive effects of repetitive anxiogenic stressor (AS) exposure on the percentages (mean ± SEM) of time spent in REM sleep for each hour of the 6 h sleep-wake recording period immediately following experimental treatment**. The treatments, listed chronologically: no exposure to AS baseline day (BLD), day 1 of AS exposure (ASD-1), day 3 of AS exposure (ASD-3), day 4 of AS exposure (ASD-4), day 5 of AS exposure (ASD-5), and no exposure to AS day (NASD). *Represents a significant difference from BLD: *p < 0.05, **p < 0.01, ***p < 0.001. Δ represents a significant difference from ASD-1: Δ*p* < 0.05, ΔΔ*p* < 0.01, ΔΔΔ*p* < 0.001.

The effects of shock administration on the percentages of time spent in REM sleep per hour are depicted in Figure 5. A two-way ANOVA indicated a significant main effect of treatment [*F*(4, 225) = 11.80, *p* < 0.0001], time [*F*(5, 225) = 4.50, *p* = 0.0006], and treatment × time interaction [*F*(20, 225) = 3.78, *p* < 0.0001]. The detailed results of *post hoc* analyses (*t*-tests) on the total percentages of time spent in REM sleep are also presented in Figure 5. These results show that REM sleep on ASD-1 was markedly reduced in the 1 h period and significantly reduced in the 2, 3, 4, 5, and 6 h periods compared to the BLD. The percentages of time spent in REM sleep on ASDs-3–5 were comparable with each other (as with W%, SWS%, and tS-R%). Also, these REM% values were significantly greater than those of the BLD in the 1 and 2 h periods of recording, and significantly greater than those of ASD-1 throughout the 6 h recording. These collective results indicate that shock treatment imparted a large, treatment day-specific effect on the amount of REM sleep, which suggests two very different behavioral responses. Additionally, REM sleep was uniquely affected during the first 2 h of the post-treatment S-W period on ASDs-3–5, compared to the final 4 h of recording. Specifically, the polygraphic signs of sleep revealed the presence of muscle tone during the first 2 h, unlike during the final 4 h, which resembled baseline-like REM sleep with complete muscle atonia.

*Post hoc* comparisons involving the NASD, compared to ASD-1, revealed an increase in the percentage of time spent in REM sleep consistent throughout the 6 h of recording (significantly higher in the 2 h period). Compared to the BLD, REM% on NAS day was also higher during the first 2 h of recording, but then reduced during the last 4 h (significantly lower in the 4 and 6 h periods). REM% on the NASD was also consistently lower than on ASDs-3–5. Collectively, these results portray a unique behavioral response that suggests that cessation of shock treatment alleviates some of the ASID-induced disturbances to REM sleep, but also that traces of these negative effects remain and require more than 2 days of relief to be eradicated.

### Effects on the latency of sleep and number of REM sleep episodes

**Figure 6:**
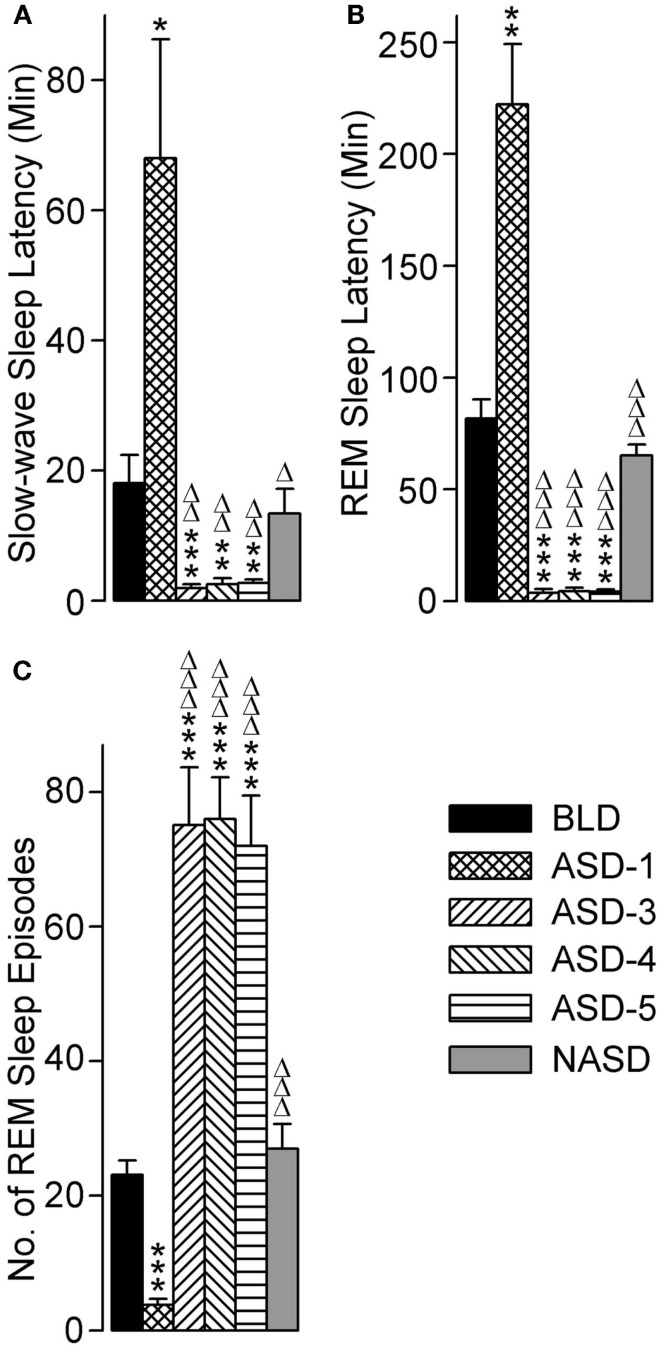
**Progressive effects of repetitive anxiogenic stressor (AS) exposure on (A) slow-wave sleep latency, (B) REM sleep latency, and (C) total number of REM sleep episodes**. Abbreviations: BLD, no exposure to AS baseline day; ASD-1, day 1 of AS exposure; ASD-3, day 3 of AS exposure; ASD-4, day 4 of AS exposure; ASD-5, day 5 of AS exposure; NASD, no exposure to AS day. *Represents a significant difference from BLD: *p < 0.05, **p < 0.01, ***p < 0.001. Δ represents a significant difference from ASD-1: Δ*p* < 0.05, ΔΔ*p* < 0.01, ΔΔΔ*p* < 0.001.

The effects of repeated shock exposure were also evaluated by measuring the latencies to the onset of both SWS and REM sleep, as well as the total number of REM sleep episodes (Figure 6). One-way ANOVA revealed a significant treatment effect on SWS latency [*F*(5, 48) = 9.869, *p* < 0.0001], REM sleep latency [*F*(5, 46) = 47.71, *p* < 0.0001], and the number of REM sleep episodes [*F*(5, 49) = 30.31, *p* < 0.0001].

Compared with the BLD (*post hoc*), the mean latency to the onset of SWS was significantly longer on ASD-1, significantly shorter on ASDs-3–5 (which were comparable with each other), and comparable on the NASD. These results illustrate a major response to the shock treatment, and one that is highly dependent on whether it is a first-time exposure or re-exposure.

The results for REM sleep latency were strikingly similar to those of SWS latency: compared with the BLD, the mean latency to the onset of REM sleep was significantly longer on ASD-1, significantly shorter on ASDs-3–5 (which were comparable with each other), and comparable on the NASD. However, the levels of statistical significance in these REM sleep latency differences were much greater than the differences in SWS latency.

Compared to the BLD, the number of REM sleep episodes was significantly less on ASD-1, significantly greater on ASDs-3–5 (which were comparable with each other), and comparable on the NASD. These results indicate that the frequency of REM sleep is also impacted by shock treatment and this impact is treatment day-specific.

### Effects on the spectral EEG power

The effects of repeated exposure to foot-shock on relative EEG power in the theta and delta bands during W, SWS, and REM sleep in the 6 h post-treatment S-W recording periods of each experimental day were also examined. Two-way ANOVAs revealed no significant main effect of treatment, time, or treatment × time interaction on relative EEG power in either spectral band during any S-W stage. Similarly, *post hoc* analyses of these data revealed no significant differences between treatment days (data not presented).

## Discussion

A number of the emotional, mental, and physical symptoms of depression can be associated with S-W disturbances (Harrison and Horne, 2000; Wagner et al., 2001;Smith and Haythornthwaite, 2004; Walker and van der Helm, 2009). Common S-W disturbances in depression include a decrease in REM sleep latency, greater frequency of REM sleep earlier in the night, sporadic bouts of wakefulness (W), early morning awakenings, reduced duration of stage 3 and stage 4 SWS, and increased duration of stage 1 SWS (Coble et al., 1976; Kupfer, 1976, 1978; Gillin et al., 1979; Reynolds and Kupfer, 1987; Sharpley and Cowen, 1995). The principal findings of this study are: (1) ASD-1 increased the total percentage of time spent in W, SWS latency, and REM sleep latency; and decreased total percentages of time spent in SWS, tS-R, and REM sleep, and the number of REM sleep episodes. (2) ASD-2 eliminated sleep altogether (100% W). (3) ASDs-3–5 decreased the total percentage of time spent in W, SWS latency, and REM sleep latency; and increased total percentages of time spent in tS-R and REM sleep, and the number of REM sleep episodes. (4) On NASD, the total percentage of time spent in SWS, SWS latency, REM sleep latency, and the number of REM sleep episodes all reverted back to BLD levels; the total percentages of time spent in W and tS-R remained at decreased levels similar to ASDs-3–5; and the total percentages of time spent in REM sleep decreased to levels between those of ASD-1 and the BLD. Collectively, these results partly validate the ASID paradigm used in the present study as a suitable animal model for studying the sleep phenotype of depression, in so far as this model’s validity depends on continued stressor exposure. However, a caveat of this interpretation is that, in the present study, we did not directly measure the behavioral signs of depression. Thus, a future study is needed to verify that the ASID model induces depression similar to other animal models of depression.

The results of the present study show that after ASD-1, rats exhibited increases in the amount of time spent in W, which were consistent throughout the entire 6 h S-W period. This increase in W was accompanied by increases in the latencies of SWS and REM sleep and decreases in the amount of time spent in SWS, tS-R, and REM sleep. On ASD-2, the loss of sleep was even more pronounced. The loss of sleep and increase in W demonstrated on these 2 days are typical characteristics of anxiety in both humans and animals (Monti and Monti, 2000; Lavie, 2001; Ohayon and Roth, 2003; Maclean and Datta, 2007; Pawlyk et al., 2008; Sanford and Yang, 2010; Macone et al., 2011; Yang et al., 2011). Contrary to the results of the present study, the previous studies that examined S-W changes associated with the LH model showed that SWS increases after the first day of LH training (Adrien et al., 1991; Fogel et al., 2011). This discrepancy could be explained by the fact that, as explained in the Introduction, these two prior studies incorporated learning components, and learning and memory has been shown to increase sleep (Karni et al., 1994; Smith, 1995; Stickgold et al., 2000; Walker et al., 2002; Huber et al., 2004). However, a future study is necessary to confirm this interpretation.

Contrary to ASD-1, ASDs-3–5 produced reductions in W, as well as corresponding decreases in SWS latency and REM sleep latency. There were also significant increases in both the amount of time spent in REM sleep, during the first 2 h, and number of REM sleep episodes. Interestingly, these S-W changes are typical of those observed in human depression (Coble et al., 1976; Garcia-Rill et al., 2008). These very similar S-W profiles on ASDs-3–5 indicate a stabilized and sustained level of S-W activity resembling that of human depression. However, to our surprise, 2 days after the last AS session when animals were not re-exposed to the AS, the ensuing S-W activity reflected a very different sleep profile. Specifically, the amount of time spent in SWS, SWS latency, REM sleep latency, and the number of REM sleep episodes all returned to BLD levels. Interestingly, REM sleep during the first 2 h of recording also returned to within range of BLD levels, yet it was significantly decreased during the latter portion of the 6 h S-W period. Therefore, lack of re-exposure to the AS yielded S-W parameters that did not resemble normal baseline sleep or the S-W phenotype typical of ASID. This indicates that AS effectively produced a depression-like sleep phenotype and that anxiety is a necessary precursor of depression in rats.

In evaluating the significant outcome of this study, we acknowledge that certain aspects of the present study could be viewed as major limitations. The first of which is the fact that the present study did not employ a triadic design. However, the need for this specific design was mitigated, as the objective of this study was to examine changes in S-W activity within the same animal. Thus, baseline S-W activity for a specific animal served as the control S-W data for that individual animal and was then used to compare with that same animal’s S-W activity after exposure to an AS. Also, a potential limitation inherent in sleep studies that employ only a single experimental group is the fact that S-W patterns over time are subject to sequential dependencies. However, this variable was negated in the present study during the habituation recording sessions during which rats’ S-W activity stabilized (Datta, 2000). This ensured little-to-no day-to-day variability in S-W patterns, allowing us to isolate the AS treatment-associated S-W changes. Finally, one additional caveat of this study, as explained earlier, is the fact that, other than S-W activity, no behavioral signs of depression were measured to confirm depression in ASID rats.

In summary, the ASID paradigm produces a sleep phenotype similar to that observed in humans with depression. Ultimately, this model could be used to study the sleep phenotype and pathophysiological mechanisms of stress-induced depression in the rat model. However, one important factor of this model is that maintaining a depression-like sleep phenotype in rats requires continual exposure to the stressor, and that sudden cessation of AS exposure appears to alleviate the depression-like S-W changes. This in turn suggests that the identification and elimination of anxiety could be a critical step for the development of behavioral and/or pharmacological therapies for treating depression.

## Conflict of Interest Statement

The authors declare that the research was conducted in the absence of any commercial or financial relationships that could be construed as a potential conflict of interest.
